# Adaptations to the Learning Environment for Elementary School Children in Georgia during the COVID-19 Pandemic

**DOI:** 10.1007/s10826-022-02531-7

**Published:** 2023-01-24

**Authors:** Olivia A. Casimir, Sarah C. Blake, Jill V. Klosky, Julie A. Gazmararian

**Affiliations:** 1 Department of Global Health, Rollins School of Public Health, Emory University, 1518 Clifton Road, Atlanta, GA 30322 USA; 2 Department of Health Policy and Management, Rollins School of Public Health, Emory University, 1518 Clifton Road, Atlanta, GA 30322 USA; 3 Department of Behavioral, Social, and Health Education Sciences, Rollins School of Public Health, Emory University, 1518 Clifton Road, Atlanta, GA 30322 USA; 4 Department of Epidemiology, Rollins School of Public Health, Emory University, 1518 Clifton Road, Atlanta, GA 30322 USA

**Keywords:** COVID-19 pandemic, Elementary schools, Virtual learning school adaptations, Academic preparedness, Digital learning resources

## Abstract

In March 2020, the rapid spread of COVID-19 led to physical school closures across the United States. Schools quickly transitioned to a remote and/or virtual learning environment. This transition had implications for students at all levels of education, especially for those most vulnerable and school-dependent for ancillary resources. The goal of this qualitative exploratory research study was to examine how public elementary schools in Georgia adapted their learning environments for students in kindergarten through third grade during the early phase of the COVID-19 pandemic. Data collection activities included school demographic surveys, parent surveys, interviews with twelve school administrators, and six focus groups with twenty-six parents. The participants discussed schools’ preparation capabilities, implementation of learning modalities, and resources for students and families. Most school personnel described the new virtual teaching demands as a hurdle for their teachers and identified several academic consequences stemming from inadequate technology access or training, such as student absenteeism and lower teaching performance. Schools lacked appropriate preparation as well as limited resources to transition to virtual learning. The COVID-19 pandemic aggravated pre-existing education and technology resource disparities for students and families of low socio-economic status or who live in rural areas. Findings from this study provide educators with information regarding deficiencies in the learning environment and provide recommendations for ongoing academic remedial efforts. Additionally, this study provides important context for the shortcomings of the COVID-19 learning environments and highlights the need to strengthen school community infrastructure and emergency planning.

## Highlights

- School administrators and parents from four public elementary schools located in urban and rural settings reported school adaptations made during the COVID-19 pandemic
- Many K-3 teachers were unprepared to provide virtual instruction and many K-3 students attending ‘high poverty’ schools lacked access to digital resources
- Three of the four schools lacked the resource capacity to support student learning needs during the spring 2020 transition and reported student absenteeism from online classes
- Schools should prioritize issues of academic preparedness and capacity building

Beginning January 2020, the COVID-19 pandemic was declared a Public Health Emergency of International Concern by the World Health Organization, a designation previously declared for diseases such the H1N1 influenza and Ebola (Cao et al., 2020; Wilder-Smith & Osman, 2020; World Health Organization, 2020). In response to this pandemic, all elementary schools in the United States (U.S.) physically closed to reduce COVID-19 transmission (Gillespie et al., 2021). Schools switched to remote learning beginning in March 2020, affecting 57 million kindergarten -12th grade (K-12) students in the U.S. (Donohue & Miller, 2020).

COVID-19 spreads through respiratory droplets and direct contact with others, which is problematic for school settings (Gillespie et al., 2021). By design, in-person schooling consists of many close contact activities, such as sharing class items, completing peer group work, and receiving one-on-one instruction from teachers (Stevenson et al., 2009). Additionally, students with disabilities (e.g., limited mobility) may experience increased difficulty following social distancing practices (Centers for Disease Control and Prevention (U.S.), 2020). Initially, children who tested positive for COVID-19 exhibited milder symptoms compared to adults (Ciotti et al., 2020). However, after the Delta variant became predominant in summer 2021, the weekly number of COVID-19 associated hospitalizations per 100,000 children and adolescents increased (Delahoy et al., 2021).

School closures have historically resulted in academic and social consequences for students (Kuhfeld et al., 2020). In previous years, U.S. school closings in response to emergency weather events (e.g., Hurricane Katrina) jeopardized student access to school meals and affected academic progress of students who received free or reduced lunch (FRL) (Kuhfeld et al., 2020; McLoughlin et al., 2020). Additionally, 80% of children with mental and behavioral health needs currently rely on schools to provide necessary social and learning resources (Masonbrink & Hurley, 2020). COVID-19-related school closures compromised children’s access to these resources and occurred alongside COVID-19-related financial instability, which created academic and financial challenges for students and families (Lancker & Parolin, 2020). Education researchers anticipate that schools will see greater achievement gaps between students from low-income families and students from higher-income families after the pandemic (Bailey et al., 2021). Students returning in fall 2020 were projected to have only 63-68% of the reading learning gains and 37-50% of the mathematical learning gains of a typical school year (Kuhfeld et al., 2020). Second- and third- graders in U.S. school districts across 22 states fell behind in reading during spring 2020 and fall 2020 (Domingue et al., 2021). Additionally, a study examining Measures of Academic Progress (MAP) Growth test scores of 2.1 million Black, Indigenous, and people of color (BIPOC) students in grades 3-8, found that math achievement declined in fall 2020, especially for fourth and fifth graders, compared to pre-pandemic math achievement scores sampled amongst same grade students (Kuhfeld et al., 2021).

To address potential learning losses, socioemotional wellbeing declines, and reduced access to vital school services, the American Academy of Pediatrics (American Academy of Pediatrics, 2021) and CDC (Centers for Disease Control and Prevention (U.S.), 2020) recommended that schools offer in-person learning during the fall 2020 semester (Gillespie et al., 2021). In the fall of 2020, U.S. schools that reopened to in-person learning still had to prepare for the ongoing threat of COVID-19 transmission while attempting to provide students with the most robust education possible. To manage COVID-19 transmission during in-person learning, the CDC released COVID-19 mitigation and infection control guidelines for schools to implement for their students, faculty, and staff (e.g., correct mask usage, handwashing, COVID-19 screening, indoor ventilation, contact tracing, and disinfection (Centers for Disease Control and Prevention (U.S.), 2020; Gettings et al., 2021).

Like the rest of the U.S., the state of Georgia faced rapid COVID-19 spread as well. An ecological analysis of COVID-19 infection rates and mortality in U.S. counties during May 2020 found that eight counties in Georgia (two in metropolitan areas and six in rural areas) had some of the highest COVID-19 mortality rates in the country (Zhang & Schwartz, 2020). The state of Georgia declared a public health emergency due to COVID-19 on March 14, 2021 (Executive Order No. 03.14.20.01, State Regulations, 2020a) and all schools in Georgia were ordered to close beginning Wednesday, March 18, 2020 (Executive Order No. 03.16.20.01, 2020b). Along with the rest of the country, Georgia schools offered remote learning modalities, coined ‘emergency remote learning’ by Hodges et al., (2020), during the remainder of the spring 2020 semester (Marshall et al., 2020). Many of the 1,328 elementary schools in the state then reopened with in-person and/or hybrid options in the fall 2020 semester (Gettings et al., 2021; Georgia Department of Education, 2020). Out of 169 Georgia elementary schools surveyed by the CDC and Georgia Department of Health (GDPH) in the fall 2020 semester (162 of which were public schools), 65.1% required masks for teachers and staff members, 51.5% utilized improved ventilation, and 18.9% spaced their desks six or more feet apart (Gettings et al., 2021).

Beyond implementing infection mitigation strategies, Georgia K-12 schools also had to adapt other aspects of school (e.g., classes, curriculum, learning modality), which may present long-lasting academic and social challenges for students and families most dependent on school resources (Kuhfeld et al., 2021). One private elementary school in Georgia reported school-based adaptations made during the fall 2020 semester in response to the COVID-19 pandemic (Basilaia & Kvavadze, 2020). The school decreased the number and duration of virtual classes for its first and second grade students and reported little to no difficulties with technology as well as high attendance in virtual classes (Basilaia & Kvavadze, 2020). However, the study only examined data from one week of school during fall 2020 and did not account for the spring 2020 semester (Basilaia & Kvavadze, 2020). Several studies examined COVID-19-related school adaptations on a national level, such as Malkus (2020) and Slavin and Storey (2020), and some studies reported the school adaptations made in specific states, such as the Catalano et al. (2021) study in New York or the Shamburg et al. (2021) study in New Jersey. However, there is a lack of research examining the abilities of public elementary schools in Georgia to provide quality education and school-related resources to students during the COVID-19 pandemic. The purpose of this study was to examine how public elementary schools in Georgia adapted their learning environments for students in kindergarten through third grade (K-3) during the early phase of the COVID-19 pandemic. Understanding gaps in the educational system can assist with anticipating students’ needs during the remainder of the pandemic and beyond.

## Methods

### Study Design

We employed a qualitative exploratory approach to examine the learning adaptations made by public elementary schools in Georgia during the early phase of the COVID-19 pandemic. Key informant interviews with school personnel and focus groups with parents of K-3 students were conducted to answer our main research question: How did public elementary schools in Georgia adapt their learning environments for K-3 students?

The research team consisted of two professors with expertise in school-based research and qualitative methods and seven graduate students at a school of public health in Georgia. In partnership with the GDPH, the research team engaged school personnel and parents of students who attended one of the four participating public elementary schools in Georgia. We conducted interviews with school administrators and school nurses and conducted focus groups with parents who had enrolled children in the participating schools. The university’s Institutional Review Board approval was deemed unnecessary as this project was considered public health practice.

### Study Setting

On average, the four participating schools had 705 students enrolled during the fall 2020 semester (Table 1). During fall 2020, in-person learning was the predominant learning modality at all participating schools. School D had the lowest number of students enrolled (501) and had the highest percentage of in-person learners (96%) amongst all the schools. One of the schools (School C) had a majority White student population while the others had a predominantly minority student population. The school with a majority Latinx student population (School D) reported that 70% of their students spoke Spanish at home. School personnel from both School B and School D identified their schools as ‘high poverty,’ while school personnel from School A and School C did not.

**Table 1:** Overall student population characteristics

School	A	B	C	D
County location	Southeast Georgia	Southwest Georgia	Northwest Georgia	Northeast Georgia
Total # students	764	792	762	501
Geography	Urban	Rural	Rural	Urban
Free/Reduced lunch eligibility (%)	41%^a^	>95%^a^	50%^a^	91%
Students who primarily speak Spanish at home (%)	2%	2%	0%	70%
Race/Ethnicity				
White/Caucasian	43%	20%	87%	2%
Black/African American	35%	74%	1%	20%
Latinx/Hispanic	11%	2%	9%	75%
Other	11%	4%	12%	3%
Learning modality				
In-person	85%	90%	90%	96%
Virtual	15%	10%	10%	4%

^a^Data from (GDOE, 2020) reported October 6, 2020

### Recruitment and Study Population

Statewide, as of March 2020, 14.4% of enrolled K-12 students in Georgia were Asian, 38.9% were White, 37.0% were Black, and 17.1% were Ethnic Hispanic (GDOE, 2020). That year, 56.18% of K-12 students in Georgia were eligible for Free and Reduced Lunch (FRL) (GDOE, 2020). There were 485,873 K-3 students enrolled in school in Georgia during fall 2020 (GDOE, 2020). As of September 2021, 60.3% of Georgia districts were providing in-person learning, 1.9% were fully remote, and 58.4% were offering hybrid learning modalities (CDC, 2021).

Four counties in Georgia were selected by the research team based on geographical designation (e.g., rural, urban), learning modalities offered (e.g., both virtual learning, and in-person learning), FRL eligibility, and regional location (e.g., Northeast, Southwest). The GDPH identified one public elementary school in each respective county, and the research team contacted the principal at each of those schools. All contacted schools were provided with a flyer describing the study goals, target population, and participation requirements, and agreed to participate. In partnership with the principals from each school, the principal, the assistant principal, and the school nurse were recruited to participate in the interviews. Each school received a $400 incentive for participation and assistance with recruiting parents to participate in the focus group discussions. This work was supported by the Emory Covid-19 Response Collaborative, which is funded by a grant from the Robert W. Woodruff Foundation.

Schools were provided with parent recruitment flyers describing the study’s focus and eligibility requirements. Administrators from the four participating elementary schools recruited parents for the focus groups through school communication channels (e.g., email, Remind app), flyers, and social media (e.g., school Facebook pages). Parents were initially selected if they met the Tier 2 classification for essential workers and if they had students in grades kindergarten to 2nd grade. Tier 2 essential workers in Georgia are defined as those employed in: food, grocery, and convenience stores; non-clinical pharmacy work; food processing, production, and manufacturing; grocery manufacturing; farming; grocery and food storage, distribution, transport, and delivery; and food service, preparation, or delivery for restaurants (Georgia Department of Early Care and Learning, 2020). Upon encountering recruitment issues, the selection criteria were later expanded to encompass any working parents whose job required them to work away from home and those with children in kindergarten up to the 3rd grade. Participating parents received an electronic $75 gift card via email as an incentive for submitting a pre-interview demographic survey and participating in the focus group.

### Data Collection

Data collection activities included interviews with school personnel, a school demographic survey, focus group with parents, and a parent demographic survey. Verbal consent was obtained from all interview and focus group participants via Zoom prior to beginning data collection activities. Each of these activities are described in more detail below.

The interview guide (Table 2) addressed several key domains: background and current position responsibilities, resource identification for essential working families, mitigation strategies, personal protective equipment, communication, resources needed from GDPH or school district, transition to virtual/hybrid learning, and student-parent outreach strategies. All research team members were trained to use the interview guide, obtain participant consent, and manage and store data. During late November 2020, four pilot interviews were conducted over Zoom with school administrators and nurses at non-participating schools in Georgia. In response to feedback from the pilot interviews, the research team created separate interview guides for nurses and school administrators. The interviews were conducted and recorded on the Zoom platform with participating school personnel during December 2020 and January 2021. Each interview was led by one to two co-interviewers and one note-taker from the research team. An electronic school demographic survey was developed and covered the following topics: number of students enrolled (in total, by grade, and by modality), percentage of students FRL eligible, percentage of students who primarily speak Spanish at home, and racial/ethnic background of student population. School demographic data as well as school-wide and grade-level percentages of students in each learning modality, were collected from school administrators to allow for subgroup analysis.

**Table 2:** School personnel interview facilitator guide

Key domains	Sample questions	Sample codes
Professional background	*How long have you held this position?*	*School Details*
	*What are your responsibilities in this position?*	*Job Responsibilities*
		*Professional Background*
Impact of COVID-19	*What did your school do over the summer to prepare for the fall 2020 semester?*	*COVID Testing*
	*Please describe any efforts your school has made to determine the needs of families with younger students whose parents are essential workers*.	*COVID Impact on Child Mental*
		*Health/Well-Being*
		*Social Interactions – Child*
Student-parent outreach strategies during COVID-19	*Are you using any targeted strategies to assist younger students (K-2) who are falling behind academically or are struggling to keep up with their work due to the COVID-19 pandemic?*	*Summer 2020 Prep for Fall 2020*
		*Spring 2020 Learning Modality*
		*Fall 2020 Learning Modality*
Public health communication	*Please describe your school’s relationship with state or local public health agencies*.	*Communication from School Regarding*
		*Health Issues*
		*PH Information and Resource Needs*
		*School Relationship to PH Agencies*
Looking forward	*What additional supports could be useful for your students?*	*School determination of Student/Family Needs*
	*What additional supports could be useful for teachers and other school personnel?*	*Resources Received – Family/Child*
		*Essential Working Parents: Challenges*

Focus group guides were developed based on the learning modality of the parents’ students (virtual-only, in-person only, or hybrid). The focus group guide topics (Table 3) addressed several key domains: satisfaction with current education method, healthcare utilization during the pandemic, satisfaction with current education method, resources and barriers during the pandemic, communication with the school, and general child well-being. All research team members were trained to use the focus group guide, obtain participant consent, and manage and store data. During February-March 2021, two pilot focus groups were conducted with parents of K-3 students at non-participating schools in Georgia over Zoom. In response to feedback from the pilot focus groups, the research team adjusted the focus group guide questions to reflect clear language and more conciseness. The six focus groups with participating parents were conducted and recorded on the Zoom platform from March to April 2021. A demographic survey for parents was developed with mostly close-ended, multiple-choice questions. This survey was administered using Qualtrics (Qualtrics, 2021) and covered the following topics: parent’s ethnicity/race, parent’s marital status, parent’s gender, parent’s occupation, and parent’s medical insurance status. Parent demographic and survey data were collected from parents who participated in the focus groups to contextualize the reported experiences.

**Table 3:** Parent focus group facilitator guide

Key domains	Sample questions	Sample codes
COVID and health	*Please discuss whether you have any concerns about your child getting COVID through exposure at school*.	*Concerns of Child Getting COVID COVID Impact on Child Mental Health/Well-Being*
School choice/modality and communication	*How do you feel about the different learning modes offered by your school – virtual, in-person or hybrid?*	*Parent Input Regarding Learning Modality*
A. Perceptions of current education method	*How have you adapted when your child has had to learn virtually?*	*Major Challenges of COVID on Student Learning*
B. Communication of current education method	*Please discuss whether the school sought input from parents about their learning modality preference*.	*Attitudes Regarding Learning Modality, Adaptations to Learning Modality – Parent/Child*
Support and barriers to support	*What school-related resources have been helpful to you since the start of the COVID-19 pandemic?*	*Resources Received – Family/Child*
A. Support – what’s working well	*What resources do you STILL need to help your child at home with their schoolwork?*	*Resources Needed – Family/Child*
B. Barriers to support	*What are additional challenges to your child’s learning experiences during COVID that we have not discussed yet?*	*Essential Working Parents and Families – Student Challenges*
		*Essential Working Parents and Families: Challenges*

### Analysis

Focus groups and interviews were audio recorded and professionally transcribed for accuracy. Study team members removed identifying information from transcripts, including names and schools. Detailed notes taken during focus groups and interviews were not used in the thematic analysis, but rather used to provide further contextual information for interpretation of the data and to augment the quality of analysis. Coding and analysis of the data was conducted by all members of the research team using a directed approach to content analysis that was guided by the literature and research question (Hsieh & Shannon, 2005). We used MAXQDA 2020 to facilitate all management and coding of the qualitative data (VERBI Software, 2020).

Research team members followed the same coding and analysis procedures for both interviews and focus groups. One codebook was developed for the interviews with school administrators and one codebook was developed for the focus groups with parents. Each codebook was organized by header levels mirroring topics from the interview and focus group guides (Tables 2 and 3). These codebooks were then reviewed by team members and modified accordingly. To ensure intercoder reliability and mitigate biases, a team-based approach (MacQueen et al., 1998) was utilized whereby teams of two research members coded the data, rotated coding with other team members, and worked among the coding teams to discuss and resolve any coding discrepancies and to revise the codebook as necessary.

Themes were developed through an iterative analysis of the inductive and deductive codes that were informed by the research question (Hsieh & Shannon, 2005). Recurring experiences, concepts, and sentiments from the participants were highlighted as key findings. Using method triangulation (Carter et al., 2014), survey data from the school and parent demographic surveys were analyzed and used to provide descriptive characteristics for participants of the study as well as context for the study’s findings. Comparisons of the findings from schools were made by geographical designation and indicators of socioeconomic status (FRL eligibility and self-reported school poverty status). Findings from the interviews and focus groups were then merged to provide differing perspectives on topics such as ‘resources needed by students and families’. Data from the school nurse interviews were not included in the thematic findings because the interviewed nurses were not involved with student learning, and this study does not discuss school adaptations related to infection control and healthcare practices. Because nurses often address student health/wellness needs, our team interviewed these school personnel to gain insight on the COVID-19 adaptations made at their schools. However, during interviews, team members found that nurses were neither involved in nor knowledgeable about the planning or implementation of COVID-19 adaptations made with regard to student learning. The preliminary findings were presented to stakeholders, including school administrator participants and members from the Georgia Department of Education, to validate the accuracy of key themes (Morse et al., 2002).

## Results

### Participant Characteristics

Twelve school personnel were interviewed (Table 4). The school personnel held similar years of experience in their respective professional fields. Principals had between 20-27 years of experience in education, assistant principals had a minimum of 18 years in education, and all nurses had 10 or more years of experience. All schools had a full-time nurse on staff. The principals oversaw staffing and scheduling, assistant principals were responsible for disciplinary measures and classroom observation, and nurses oversaw student health and medicine administration. Nurses at two of the schools, Schools A and C, provided vision and/or hearing screenings to students.

**Table 4:** Interview participant characteristics average time in field and position by school and role n = 12

School	A	B	C	D
County location	Southeast	Southwest	Northwest	Northeast
	Georgia	Georgia	Georgia	Georgia
Geography	Urban	Rural	Rural	Urban
Gender				
Male	0% (0)	33% (1)	33% (1)	0% (0)
Female	100% (3)	67% (2)	67% (2)	100% (3)
Time spent in professional field (years)				
Principal	25	21	20	37
Assistant principal	18	21	25	29
Nurse	11	46	12	47
Time spent in position (years)				
Principal	7	9	4	3
Assistant principal	2.5	9	4	4
Nurse	1.5	15–18	4	5

Twenty-six parents participated in the six focus groups, which were combined for ease of data presentation (Table 5). Two to six parents attended each focus group. Two of the four schools (schools C and D) had more than one focus group. One focus group consisted of only Spanish-speaking parents. Participants were White/Caucasian (48%), Hispanic/Latinx (27%), and Black/African American (23%). The majority of the parents were female (85%), were married/partnered (69%) and had private insurance (58%). The top three occupations of participants were clinical, teaching, and housekeeping.

**Table 5:** Focus group participant characteristics six focus groups (*n* = 26)

Demographic category	Percentage reported
Race/Ethnicity (Top 3)	
White/Caucasian	46% (12)
Hispanic/Latinx	27% (7)
Black/African American	23% (6)
Gender	
Female	85% (22)
Male	15% (4)
Marital status	
Married/Partnered	69% (18)
Single	19% (5)
Divorced	12% (3)
Occupations (Top 3)	
Clinical	31% (8)
Teaching	19% (5)
Housekeeping	12% (3)
Insurance status	
Public	42% (11)
Private	39% (10)
Uninsured	4% (1)

### Thematic Findings

#### Learning options provided to students and families

##### Schools employed various strategies to prepare teachers and modify technology resources for the fall 2020-spring 2021 academic year

During summer 2020, schools spent most of the pre-planning time providing teachers with training for setting up and using online platforms (e.g., Google Classroom, Zoom, Google Meets) (Table 6). Schools A and D reported providing professional learning opportunities for technology education to their teachers and School A reported that its teachers had already been using an online platform.

“*We have a very strong technology department here … and there were a lot of trainings that were offered throughout the summer”* (Assistant Principal, School A)

“*We had done a lot already with Google Meets [pre-pandemic] and so our teachers were somewhat familiar with it.”* – (Principal, School A)

**Table 6:** Key themes

Themes	Sub-themes
Learning options provided to students and families	Schools employed various strategies to prepare teachers and modify technology resources for the fall 2020-spring 2021 academic year.
	Schools offered different learning modalities to students in response to COVID-19-related circumstances.
Challenges to learning modality implementation	Some parents and school personnel felt teachers lacked the time and preparation to teach virtually against other competing demands and felt that this negatively affected teaching performance.
*Lack of academic preparedness*	School personnel and parents felt students were most supported during face-to-face instruction and listed several shortcomings of the virtual learning modality implemented at their schools.
*Issues related to lack of technology infrastructure*	Schools reported varying levels of technological infrastructure to support the change to virtual learning.
Strategies employed to address potential academic or mental health concerns	Schools reported varying methods for addressing the academic progress of their students.
	Schools implemented several strategies to address the mental health of their students.
Resources and determination of needs outside the classroom	Schools provided and/or made parents aware of food, technology, and counseling resources but parents described barriers to utilizing these services.

Administrators from School D, the other ‘high poverty’ school, reported positive attitudes towards their school’s pre-planning preparation. This school’s district provided professional learning to their teachers and created a districtwide Zoom schedule to support families with multiple students attending classes on one device. While most schools reported similar experiences, School B, one of the ‘high poverty’ schools, reported less progress with summer preparation. For instance, one of the administrators from the school reported discontent with the county’s planning.

“*There was a lot of wasted time in the spring. I felt that at the administrative level, from the district. While other schools around us were getting grants and they were supplying students with devices, hotspot. We had Zoom meetings and they lasted for hours. And I can’t really recall any decisions that we made then…we waited until July to start training teachers how to use the Chromebooks.”* (Principal, School B)

##### Schools offered different learning modalities to students in response to COVID-19-related circumstances

In the fall of 2020, all schools offered options for both in-person and virtual learning, and most offered hybrid options as well, which allowed students to learn in-person for 2 or 3 days during the week and then learn virtually for the remaining days. Parents were allowed to select their children’s learning modality option and received opportunities to change during the school year. Some schools had stipulations for families who wanted their child(ren) to learn virtually. For instance, one school required parents to sign a contract at the beginning of the school year listing requirements for virtual learning (e.g., adult supervision during online class). In some schools, teachers only taught one modality (e.g., virtual only, in-person only) while teachers in other schools taught both modalities. For all learning modalities, teachers were available for instruction and meetings with students and parents during certain periods of the day, (e.g., from 8:00am to 1:00 pm). From March to May 2020, most schools used paper packets as mediums for learning, and organized times for parents to pick up and return the packets. All school-related extracurricular activities were discontinued during the spring 2020 and fall 2020 semesters, but schools still incorporated classes such as P.E. and art into the curriculum when students returned to in person learning in the fall.

“*… we have a Virtual Classroom Academy, which is all virtual for a semester, and then parents can choose to go back to face-to-face and those in face-to-face can choose to go to virtual after that, after a semester”* (Assistant Principal, School D)

### Challenges to Learning Modality Implementation

#### Lack of academic preparedness

##### Some parents and school personnel felt teachers lacked the time and preparation to teach virtually against other competing demands and felt that this negatively affected teaching performance

School personnel reported that some teachers were less proficient with technology, which limited the time teachers spent planning and teaching the material. School personnel felt K-3 teachers were especially unprepared to teach virtually because they did not normally use as much technology while instructing younger students, and parents echoed this sentiment.

“*When you’re teaching early elementary, your main focus is the kids who are in front of you. And that’s what you went to school for. Most of them did not go to school to be a tech support or parents to students … a lot of them found themselves in that role*.” (Principal, School B)

“*…we started out doing the virtual with them. And it honestly was such a mess that we gave up on doing it and pulled them all out and homeschooled them…I didn’t feel that the teachers were very well-prepared for what they had to deal with going to a virtual situation versus in class.”* (Parent)

##### School personnel and parents felt students were most supported during face-to-face instruction and listed several shortcomings of the virtual learning modality implemented at their schools

One administrator indicated that live interaction with the teacher allows for immediate feedback and adult supervision. Another administrator commented that it is difficult to replicate the one-on-one, hands-on instruction needed for lessons with younger students (e.g., learning letter writing) in the virtual environment. Several parents felt that remote learning instruction should have relied less on paper packets. Administrators reported that these packets were often not returned and stated that many of the returned packets were incomplete. Additionally, schools had challenges providing education for students with disabilities in a virtual environment because the individual education plans (IEPs) for these students were only designed for in-person learning. The students studying online also had to contend with challenges in the remote learning setting. There were often other family members or multiple children in the home, and the background noise was distracting for both the class and the students.

“*a 50-minute virtual segment that she’s online with those kids, so much of that time is spent trying to get them to get to the right technology, to access what you need them to access …. So, I think the actual teaching has suffered just because they don’t have as much time to impart knowledge*” (Assistant Principal, School C)

“*And the teachers, I’m telling you now, they wanted those kids back in that classroom because they knew they were not reaching them.”* (Assistant Principal, School B)

However, parents and school personnel reported that students learning in-person were also subject to disruptions to class time due to frequent, inconsistent quarantines and school closures.

#### Issues related to lack of technology infrastructure

##### Schools reported varying levels of technological infrastructure to support the change to virtual learning

Findings suggest that none of the schools were equipped to provide universal access to remote learning technology. Only one school, School A, reported having sufficient digital devices for students and teachers as well as a strong technology support in place for remote learning, while the other three schools lacked this resource capacity.

“*We have many parents who are not working… they did not have access to a device. They did not have internet, or the connectivity was so poor that they weren’t able to do a whole lot in the spring. Plus, most of our teachers who in K or second grade did not have access to Chromebooks for instruction*.” (Principal, School B)

Many students at both rural schools (Schools B and C) and one urban school (School D) had limited digital devices and/or internet connectivity. Some had to attend class from a cell phone or public place with Wi-Fi. All three schools reported low Zoom attendance during spring 2020; School D merged some of the virtual classes in the same grade level together and neither School B nor School C made virtual attendance mandatory.

#### Strategies employed to address potential academic or mental health concerns

##### Schools reported varying methods for addressing the academic progress of their students

School C reported having an academic review board and process to address cases where virtual learners struggled due to incomplete assignments or lack of attendance. The review board provided academic standards and parental supervisory requirements for a student to remain enrolled in the virtual modality and required students to either homeschool or enroll in in-person learning if those guidelines were not met. Several teachers at School D made themselves available by phone late at night to help parents with their children’s homework and to check in with students.

“*My teachers … I had them call and check in over the phone with their students, especially, those students that didn’t have access to a device … if there was anything they needed resource-wise, we could help them*.” – (Principal, School A)

The two urban schools (Schools A and D) were able to offer early intervention teachers to support in-person learners, but most schools could not offer tutoring resources beyond class instruction. Most schools noted that there was not any milestone testing, therefore schools lacked spring data to measure learning outcomes, but one school (School C) received data from the state program ‘GKIDS,’ which is a progressive evaluation conducted for kindergarteners throughout the year.

##### Schools implemented several strategies to address the mental health of their students

Many teachers noticed that their students missed physical touch (e.g., hugs, high-fives) and taught them safer alternatives such as self-hugs and finger waves.

“*But they [the students] just wanted to hug, so we did the elbow bumps and stuff instead, and they were so excited.”* (Assistant Principal, School D)

During Spring 2020, teachers at School B dressed up in animal costumes while students were picking up packets, and others drove by their students’ homes to boost morale. Teachers at School A recorded messages from teachers to be sent out as a school video to their students, and one teacher even sang to students.

“*Our kindergarten teacher that teaches our virtual kindergarten class, she has to stop every few minutes and she’ll break into song, some kind of from Frozen or something to kind of try to grasp their attention, pop up little things to try to keep them engaged*.” (Principal, School A)

Year-long, all schools had a counselor available for their students and offered more extensive resources for mental/behavioral health to their students at the county level. Some schools provided staff with training to identify warning behaviors (e.g., aggression, withdrawal) in children for potential mental health challenges. One school (School A) reported that, before the pandemic, they had already been working on becoming a “trauma-informed school.” The counselor at School D held group meetings with students who experienced family loss from COVID-19. But some parents wished the counselors reached out to students to set up more individual or group meetings with students to address mental health needs and to maintain peer social engagement.

#### Resources and determination of needs outside the classroom

##### Schools provided and/or made parents aware of food, technology, and counseling resources but parents described barriers to utilizing these services

Schools provided free breakfast and lunch, limited Chromebooks and/or laptops for temporary use, online learning platforms for reading and math, and district-level aid for extraordinary circumstances such as homelessness. Three of the schools reported that county level staff conducted home visits to gauge the needs of at-risk families. School D provided in-person computer training to select parents and School B’s county-level parent coordinator held classes for parents on pertinent topics such as technology use.

Parents reported barriers to accessing these resources, such as lack of connectivity in rural areas, lack of childcare options, time-conflicting work schedules, and lack of parent proficiency with technology. Although schools called and emailed parents when food resources were available at local pantries, parents were sometimes working during the food distribution hours. While some schools provided Wi-Fi spots in public spaces, parents felt the schools should have done more to improve student access to internet and digital devices. In addition to internet connectivity, parents also desired more parent education and technology use resources from their children’s schools.

“*…being in a small town, I pay $150 a month for internet that may or may not work*.” (Parent)

“*I’d like for us parents to have classes on English or on how to use computers … I think that if we, as parents, knew a little more, we could help our children a little more*.” (Parent who speaks Spanish only, translated from Spanish)

## Discussion

This study examined how public elementary schools in Georgia adapted their learning environments for students in kindergarten through third grade during the early phase of the COVID-19 pandemic. Findings suggest that schools offered different learning modalities to students and implemented various strategies to address students’ academic and mental health. Schools continued offering meals to all students, but some schools lacked the resource capacity to support student learning needs during spring 2020. Some K-3 teachers were unprepared to provide virtual instruction, and many K-3 students attending the ‘high poverty’ schools did not have the digital resources required to engage in virtual classes.

During the early phase of the COVID-19 pandemic, most K-12 schools in the United States were unprepared to adjust their learning environment (Francom et al., 2021). Consistent with our findings about teacher training needs during summer 2020 pre-planning, Kennedy (2018) and Marshall et al. (2020) reported that K-12 teachers tended to have less familiarity with technology and had to quickly learn how to utilize online educational platforms. A comparative analysis of common search terms and content use on a global education support website during February-March 2020 found that educators most sought content about setting up, providing lessons, and communicating in the digital learning environment (Cavanaugh & DeWeese, 2020). However, two large independent K-12 schools in the U.S. (one in the South, one in the Mid-Atlantic) in the Gillespie et al. (2021) study were better prepared to continue school during the COVID-19 pandemic. Unlike the schools in this study, both independent schools offered on-campus COVID-19 testing, tested all their students and staff after Thanksgiving Break, and reported having contingency plans for the transition to virtual learning (Gillespie et al., 2021).

Three other studies in schools (Nasrullah et al., 2012; Francom et al., 2021; Fox, 2004) encountered classroom and resource challenges during infectious disease outbreaks as well. Consistent with our findings regarding COVID-19-related school adaptations, many of the 704 surveyed Georgia public schools in the Nasrullah et al. (2012) study held shorter school days and had student absenteeism during the H1N1 pandemic. However, diverging from our findings, schools did not transition to remote learning or discontinue school activities, and most did not experience greater than one week with significant respiratory illness or absenteeism amongst students (Nasrullah et al., 2012). Like the school districts in our study, the Francom et al., (2021) study revealed that during COVID-19, Mississippi and South Dakota school districts also provided their teachers with varying levels of training and support for distance teaching. Departing from our findings about insufficient digital devices, 62% of the surveyed teachers reported that their school was able to provide a digital device to each student (Francom et al., 2021). Similar to teachers from Schools B and C, teachers who taught in Hong Kong during SARS epidemic school closures (Fox, 2004; Francom et al., 2021) reported student absenteeism during online classes. Similar to teachers from Schools A and D, those teachers received professional technology education, yet still reported persisting barriers to effective online instruction including lack of practical training for online teaching (Fox, 2004; Francom et al., 2021).

Students from low-income families often depend on several school-based services (e.g., temporary computer use, access to school meals, mental health services and rehabilitation therapies) and thus had lower capacity to navigate the COVID-19-related school closures (Donohue & Miller, 2020; Masonbrink & Hurley, 2020). An Education Trust poll, which surveyed U.S. parents, indicated that 50% of low-income families and 42% of families of color did not have access to the digital devices and/or internet necessary to accommodate distance learning (The Education Trust & Global Strategy Group, 2020). Additionally, 2-5% of students in U.S. public schools have unstable housing, which is another obstacle to distance learning (Engzell et al., 2021). Schools B and D in our study reported similar experiences; both schools self-identified themselves as high poverty schools, reported Black-majority or Latinx-majority student populations with at least 90% of students FRL-eligible (GDOE, 2020), respectively, and described difficulties providing sufficient devices and internet to students and teachers. Furthermore, although schools provided online learning during the COVID-19 pandemic, high-poverty schools had a higher proportion of students absent from these online lessons compared to low-poverty schools (Kuhfeld et al., 2020). This was consistent with one of this study’s findings since both ‘high-poverty’ schools in our study (Schools B and D) reported high student absenteeism during spring 2020. However, our study also diverged from these findings in two ways: (1) School C, a ‘non-high poverty’ school with the second-lowest percentage of students FRL-eligible (50%), also reported student absenteeism, and (2) our study found that student absenteeism during virtual spring 2020 classes was linked to both rural schools in the study.

Schools continued to provide lunch as a resource to students attending remote learning during the spring of 2020 due to a waiver granted by the U.S. Department of Agriculture (McLoughlin et al., 2020). Consistent with the schools in our study, other public schools in large urban school districts outside of Georgia, such as the Houston Independent School District (HISD) and Chicago Public school district, employed methods to provide food resources directly and indirectly to students and families (McLoughlin et al., 2020). These methods included providing families with meal boxes and timely referrals to food resources (e.g., local food pantries) (McLoughlin et al., 2020). However, departing from the schools in our study, these school districts formed partnerships with local health/relief organizations to increase resource capacity and facilitate meal distribution to their students (McLoughlin et al., 2020). For instance, the HISD partnered with the police department and a local food bank to aid food distribution (McLoughlin et al., 2020). The New York City Department of Education provided hot meals to essential working parents’ children and partnered with Door Dash to deliver meals to medically vulnerable students (McLoughlin et al., 2020). Compared to these schools, the schools in our study were unable to offer the same level of targeted resource support to these students.

Based on the school challenges experienced during the spring and fall 2020 semesters, many parents and school personnel in our study felt face-to-face learning was the best mode of instruction due to more effective teaching and peer interaction. However, the transition to virtual learning was born out of the circumstances of the COVID-19 pandemic, which did not allow sufficient time for proper planning and resource allocation for virtual learning and instruction (Hodges et al., 2020; Marshall et al., 2020). Some researchers contend that when properly implemented, virtual learning has the potential to offer increased learning diversity and supplemental instruction to students with different learning styles or with special needs (Hasselbring & Glaser, 2000; Kennedy, 2018). Notably, half the teachers in the Francom et al., (2021) study planned to continue using online learning management systems (e.g., Remind, Zoom, Google Meet) in conjunction with face-to-face instruction to increase capacity for parent communication, virtual field trips/guest speakers, and participation for absent students (Francom et al., 2021). This is an important consideration when deciding what learning modalities should continue to be offered to students in the future.

### Strengths

This study has several strengths. First, because this was a qualitative study, there were rich sources of data that supported our findings regarding schools’ adaptations to the COVID-19 pandemic. The participants’ perceptions and experiences presented context for some of the COVID-19 response and learning environment changes seen during the spring 2020 and fall 2020 semesters. Second, because both parents of K-3 students and school personnel of those students’ schools were interviewed for this study, data captured different perspectives of the same issues. Third, the same type of school personnel was interviewed at each school allowing for equitable comparison of their perceptions.

### Limitations

There are also several limitations of our study that warrant discussion. First, this study did not interview teachers due to the intensity of their teaching workload during the COVID-19 pandemic, so the findings related to challenges and perceptions of teachers are secondary accounts. Second, because of the nature of qualitative work, the findings are not representative of the experiences and COVID-19 response of all public elementary schools in Georgia. Third, although the study recruited essential working parents to discuss their perceptions of the COVID-19 learning environment, the interviewed school personnel only discussed strategies utilized for all K-3 students in their school because schools did not single out data from students of essential working parents. Therefore, the findings do not solely represent the learning experiences of students of essential working parents. Fourth, the subgroup analysis comparing socioeconomic status amongst the schools relies only on free/reduced lunch eligibility and self-report rather than more reliable demographic characteristics, which limits the accuracy of those comparisons.

## Conclusion and Future Research Directions

The COVID-19 pandemic has continued to present widespread unprecedented changes to the educational system (McLoughlin et al., 2020) and this study’s findings remain extremely relevant. While the weekly rate of new U.S. cases has declined from the summer 2021 COVID-19 surge (from 1,020,072 incident cases in late August 2021 to 550,684 incident cases in mid-November 2021), students may have to experience remote learning again during potential future surges (CDC, 2020; WHO, 2021a; WHO, 2021b).

Moving forward, it will be important to create well-designed, sustainable infrastructure to support education in future emergencies by school districts prioritizing issues of emergency preparedness and capacity building. School districts should strengthen their technology support that would enable students, teachers, and families to provide or receive education through online platforms and digital devices. Schools might also consider the benefits of incorporating more comprehensive health resources and education on campus to reduce infectious disease transmission amongst elementary students. For instance, prior to the pandemic, one community with limited nursing resources permitted nursing students to teach elementary students 20-minute lessons on handwashing and hand hygiene (Perry et al., 2021). Finally, it is important to note that during COVID-19, education disparities increased for Black, Hispanic/Latino, and American Indian/Alaska Native children, and children who are learning English, have disabilities, or are living in poverty (AAP, 2021). Given that schools are an integral part of children’s support system, public health entities need to mobilize partnerships to provide learning, food, mental health, and childcare resources to students and families affected by school closures or living with parents/guardians experiencing illness (Stevenson et al., 2009). School districts should build their capacity to support low-income, rural, and disabled students in order to reduce educational disparities that have become more prevalent during the pandemic.

Future research should seek the voices of more stakeholders involved in education to gauge areas of need for the federal government, state entities, and school districts to address. It will be invaluable to obtain the perspectives of elementary school teachers, who experienced first-hand accounts of the learning environment before and during the COVID-19 pandemic. Counselors can speak about ways their schools utilized their role to address students’ emotional needs during the COVID-19 pandemic, as well as changes in student mental health, and resources needed. Because the research team encountered recruitment issues, subsequent studies should examine barriers and facilitators to engaging essential working parents in school-related surveys. Moving forward, research might further explore the requirements and benefits of effective implementation of alternative learning modalities (e.g., periodic virtual learning, flipped classroom). Future studies may also examine the implications of the approval of the COVID-19 vaccine for elementary-aged children on the availability of student learning options (Kozlov, 2021). The findings from this study about lack of academic preparedness and resource capacity for low-income families or rural communities may encourage further research into solutions for technology and education disparities between schools from different regions and income levels.

## References

[CR1] American Academy of Pediatrics. (2021). *Covid-19 guidance for safe schools and promotion of in-person learning*. https://www.aap.org/en/pages/2019-novel-coronavirus-covid-19-infections/clinical-guidance/covid-19-planning-considerations-return-to-in-person-education-in-schools/.

[CR2] Bailey DH, Duncan GJ, Murnane RJ, Au Yeung N (2021). Achievement gaps in the wake of Covid-19. Educational Researcher.

[CR3] Basilaia, G., & Kvavadze, D. (2020). Transition to online education in schools during SARS-CoV-2 coronavirus (Covid-19) pandemic in Georgia. *Pedagogical Research*, *5*(4). 10.29333/pr/7937.

[CR4] Cao Y, Cai K, Xiong L (2020). Coronavirus disease 2019: a new severe acute respiratory syndrome from Wuhan in China. Acta Virologica.

[CR5] Carter N, Bryant-Lukosius D, DiCenso A, Blythe J, Neville AJ (2014). The use of triangulation in qualitative research. Oncology Nursing Forum.

[CR6] Catalano AJ, Torff B, Anderson KS (2021). Transitioning to online learning during the Covid-19 pandemic: differences in access and participation among students in disadvantaged school districts. International Journal of Information and Learning Technology.

[CR7] Cavanaugh C, DeWeese A (2020). Understanding the professional learning and support needs of educators during the initial weeks of pandemic school closures through search terms and content use. Journal of Technology and Teacher Education.

[CR8] Centers for Disease Control and Prevention (U.S.). (2020). *Guidance for Covid-19 prevention in K-12 schools*. https://www.cdc.gov/coronavirus/2019-ncov/community/schools-childcare/k-12-guidance.html. 34009772

[CR9] Centers for Disease Control and Prevention (U.S.). (2021). *Covid-19-related school closures and learning modality changes—United States, August 1-September 17, 2021*. https://stacks.cdc.gov/view/cdc/109969. 10.15585/mmwr.mm7039e2 PMC8486392 34591828

[CR10] Ciotti M, Ciccozzi M, Terrinoni A, Jiang WC, Wang CB, Bernardini S (2020). The Covid-19 pandemic. Critical Reviews in Clinical Laboratory Sciences.

[CR11] Delahoy, M. J., Ujamaa, D., Whitaker, M., O’Halloran, A., Anglin, O., Burns, E., Cummings, C., Holstein, R., Kambhampati, A. K., Milucky, J., Patel, K., Pham, H., Taylor, C. A., Chai, S. J., Reingold, A., Alden, N. B., Kawasaki, B., Meek, J., & Yousey-Hindes, K., COVID-NET Surveillance Team. (2021). Hospitalizations associated with Covid-19 among children and adolescents – COVID-NET, 14 States, March 1, 2020-August 14, 2021. *MMWR. Morbidity and Mortality Weekly Report*, *70*(36), 1255–1260. 10.15585/mmwr.mm7036e2. 10.15585/mmwr.mm7036e2 PMC8437052 34499627

[CR12] Domingue, B. W., Hough, H. J., Lang, D., & Yeatman, J. (2021). *Changing patterns of growth in oral reading fluency during the Covid-19 pandemic*. Policy Analysis for California Education, PACE. https://edpolicyinca.org/publications/changing-patterns-growth-oral-reading-fluency-during-covid-19-pandemic.

[CR13] Donohue JM, Miller E (2020). Covid-19 and school closures. Journal of the American Medical Association.

[CR14] The Education Trust & Global Strategy Group. (2020). *Parents overwhelmingly concerned their children are falling behind during school closures*. The Education Trust. https://edtrust.org/parents-overwhelmingly-concerned-their-children-are-falling-behind-during-school-closures/.

[CR15] Engzell, P., Frey, A., & Verhagen, M. D. (2021). Learning loss due to school closures during the Covid-19 pandemic. *Proceedings of the National Academy of Sciences of the United States of America*, *118*(17). 10.1073/pnas.2022376118. 10.1073/pnas.2022376118 PMC8092566 33827987

[CR16] Executive Order No. 03.14.20.01, State Regulations. (2020a). https://gov.georgia.gov/executive-action/executive-orders/2020-executive-orders.

[CR17] Executive Order No. 03.16.20.01, State Regulations. (2020b). https://gov.georgia.gov/executive-action/executive-orders/2020-executive-orders.

[CR18] Fox, R. (2004). SARS epidemic: Teachers’ experiences using ICTs. *Beyond the Comfort Zone**:**Proceedings of the 21st ASCILITE Conference*, 319–327. https://www.ascilite.org/conferences/perth04/procs/fox.html.

[CR19] Francom, G. M., Lee, S. J., & Pinkney, H. (2021). Technologies, challenges and needs of K-12 teachers in the transition to distance learning during the Covid-19 pandemic. *TechTrends: For Leaders in Education & Training*, 1–13. 10.1007/s11528-021-00625-5. 10.1007/s11528-021-00625-5 PMC8233184 34223560

[CR20] Gettings J, Czarnik M, Morris E, Haller E, Thompson-Paul AM, Rasberry C, Lanzieri TM, Smith-Grant J, Aholou TM, Thomas E, Drenzek C, MacKellar D (2021). Mask use and ventilation improvements to reduce Covid-19 incidence in elementary schools – Georgia, November 16-December 11, 2020. MMWR: Morbidity & Mortality Weekly Report.

[CR21] Georgia Department of Early Care and Learning. (2020). Essential services workforce priority group – Georgia. https://caps.decal.ga.gov/assets/downloads/COVID-19_Priority_Group_Memorandum_4.1.2020.pdf.

[CR22] Georgia Department of Education. (2020). Covid-19 (Coronavirus) data & reporting. https://www.gadoe.org/Pages/Home.aspx.

[CR23] Gillespie DL, Meyers LA, Lachmann M, Redd SC, Zenilman JM (2021). The experience of 2 independent schools with in‐person learning during the Covid‐19 pandemic. The Journal of School Health.

[CR24] Hasselbring TS, Glaser CH (2000). Use of computer technology to help students with special needs. The Future of Children.

[CR25] Hodges, C., Moore, S., Lockee, B., Trust, T., & Bond, A. (2020). *The difference between emergency remote teaching and online learning*. Educause Review. https://er.educause.edu/articles/2020/3/the-difference-between-emergency-remote-teaching-and-online-learning.

[CR26] Hsieh HF, Shannon SE (2005). Three approaches to qualitative content analysis. Qualitative Health Research.

[CR27] Kennedy, K. (2018). *Handbook of research on K-12 online and blended learning* (2nd ed.). Carnegie Mellon University. 10.1184/R1/6686813.v1.

[CR28] Kozlov M (2021). What Covid vaccines for young kids could mean for the pandemic. Nature.

[CR29] Kuhfeld M, Soland J, Tarasawa B, Johnson A, Ruzek E, Liu J (2020). Projecting the potential impact of Covid-19 school closures on academic achievement. Educational Researcher.

[CR30] Kuhfeld, M., Ruzek, E., Lewis, K., Soland, J., Johnson, A., Tarasawa, B., & Dworkin, L. (2021). *Understanding the initial educational impacts of Covid‐19 on communities of color*. NWEA Research. https://www.nwea.org/content/uploads/2021/06/Understanding-the-initial-educational-impacts-of-COVID19-on-communities-of-color.Resear-hBrief.pdf.

[CR31] Lancker WV, Parolin Z (2020). Covid-19, school closures, and child poverty: a social crisis in the making. The Lancet.

[CR32] MacQueen KM, McLellan E, Kay K, Milstein B (1998). Codebook development for team-based qualitative analysis. Cultural Anthropology Methods.

[CR33] Malkus, N. (2020). Reopening in the shadow of Covid-19: beginning the first full coronavirus school year. *American Enterprise Institute*. https://www.jstor.org/stable/resrep26789.

[CR34] Marshall DT, Shannon DM, Love SM (2020). How teachers experienced the Covid-19 transition to remote instruction. Phi Delta Kappan.

[CR35] Masonbrink, A. R., & Hurley, E. (2020). Advocating for children during the Covid-19 school closures. *Pediatrics*, *146*(3). 10.1542/peds.2020-1440. 10.1542/peds.2020-1440 32554517

[CR36] McLoughlin GM, McCarthy JA, McGuirt JT, Singleton CR, Dunn CG, Gadhoke P (2020). Addressing food insecurity through a health equity lens: a case study of large urban school districts during the Covid-19 pandemic. Journal of Urban Health.

[CR37] Morse JM, Barrett M, Mayan M, Olson K, Spiers J (2002). Verification strategies for establishing reliability and validity in qualitative research. International Journal of Qualitative Methods.

[CR38] Nasrullah M, Breiding MJ, Smith W, McCullum I, Soetebier K, Liang JL, Drenzek C, Miller JR, Copeland D, Walton S, Lance S, Averhoff F (2012). Response to 2009 pandemic influenza A H1N1 among public schools of Georgia, United States—fall 2009. International Journal of Infectious Diseases.

[CR39] Perry J, McClure N, Palmer R, Neal JL (2021). Utilizing academic-community partnerships with nursing students to improve hand hygiene in elementary students to reduce transmission of Covid-19. NASN School Nurse.

[CR40] Qualtrics. (2021). *Version March 2020 [computer software]*. Provo, UT, USA: Qualtrics. https://ww.qualtrics.com.

[CR41] Shamburg C, Amerman T, Zieger L, Bahna S (2021). When school bells last rung: New Jersey schools and the reaction to Covid-19. Education and Information Technologies.

[CR42] Slavin RE, Storey N (2020). The US educational response to the Covid-19 pandemic. Best Evidence in Chinese Education.

[CR43] Stevenson E, Barrios L, Cordell R, Delozier D, Gorman S, Koenig LJ, Odom E, Polder J, Randolph J, Shimabukuro T, Singleton C (2009). Pandemic influenza planning: addressing the needs of children. American Journal of Public Health.

[CR44] VERBI Software. (2020). *MAXQDA 2020 [computer software]*. Berlin, Germany: VERBI Software. https://maxqda.com.

[CR45] Wilder-Smith A, Osman S (2020). Public health emergencies of international concern: a historic overview. Journal of Travel Medicine.

[CR46] World Health Organization. (2020). *Covid-19 public health emergency of international concern (PHEIC) Global research and innovation forum: towards a research roadmap*. Global Research Collaboration for Infectious Disease Preparedness. https://www.who.int/publications/m/item/covid-19-public-health-emergency-of-international-concern-(pheic)-global-research-and-innovation-forum.

[CR47] World Health Organization. (2021a). *Covid-*19 we*ekly epidemiological update – 24 August 2021*. World Health Organization. https://www.who.int/publications/m/item/weekly-epidemiological-update-on-covid-19---24-august-2021.

[CR48] World Health Organization (2021b). *Covid-19 weekly epidemiological update – 16 November 2021*. World Health Organization. https://www.who.int/publications/m/item/weekly-epidemiological-update-on-covid-19---16-november-2021.

[CR49] Zhang, C. H. & Schwartz, G. G. (2020). Spatial disparities in coronavirus incidence and mortality in the United States: an ecological analysis as of May 2020. *The Journal of Rural Health: Official Journal of the American Rural Health Association and the National Rural Health Care Association*, *36*(3), 433–445. 10.1111/jrh.12476. 10.1111/jrh.12476 PMC7323165 32543763

